# Quantitative proteome dataset profiling of *UBC4* and *UBC5* deletion strains in *Saccharomyces cerevisiae*

**DOI:** 10.1016/j.dib.2022.108737

**Published:** 2022-11-09

**Authors:** Valentina Rossio, Joao A Paulo

**Affiliations:** Department of Cell Biology, Blavatnik Institute at Harvard Medical School, Boston, MA 02115, USA

**Keywords:** Ubiquitin proteasome system, Protein stability, Yeast, TMT, Isobaric tagging

## Abstract

The Ubiquitin-Proteasome System (UPS) regulates many cellular processes in eukaryotic cells. Ubiquitylation by the UPS mainly directs proteins to proteasomal degradation, but it can also have non-degradative functions, such as regulating protein activity or localization. The small protein ubiquitin is conjugated to its substrates via a cascade of E1-E2-E3 enzymes. Dysregulation of the UPS has been implicated in the genesis and progression of many diseases, such as neurodegenerative diseases and cancer; thus, the UPS components are attractive targets for developing pharmaceutical drugs. E2s, or ubiquitin conjugating enzymes, are central players of the UPS. E2s function in tandem with specific ubiquitin ligases (E3s) to transfer ubiquitin to substrates. Here, we present the first proteome stability analysis of two closely related ubiquitin conjugating enzymes, Ubc4 and Ubc5, in *S. cerevisiae*. These two E2s are nearly identical, having 92% sequence identity and differing by only 11 amino acid residues. This dataset is of broad interest because higher eukaryotes express ubiquitin conjugating enzymes that are analogous to the yeast Ubc4/5. The data have been deposited in ProteomeXchange with the dataset identifier PXD037315.

**Specifications Table**

**Table:** 

Subject	Omics: Proteomics
Specific subject area	Yeast, proteomics, Ubiquitin Proteasome System
Type of data	Figure Excel Table RAW mass spectrometry data files
How the data were acquired	Mass spectrometric data were collected on an Orbitrap Eclipse mass spectrometer coupled to a Proxeon NanoLC-1200 UHPLC (ThermoFisher Scientific). Data were acquired using Xcalibur 4.1 and Tune 3.5. RAW files were converted to mzXML using MSconvert 3.0. Database searches were performed with the Comet (2022.01 rev. 0) search engine.
Data format	Raw Analyzed
Description of data collection	Exponentially growi*ng S. cerevisiae* (wild type, *ubc4Δ* and *ubc5Δ*) cells were collected. Proteins were chloroform-methanol precipitated and digested with LysC followed by trypsin, prior to LC-MS/MS analysis. Digested samples were labeled with tandem mass tag (TMTpro) reagents. The pooled sample was fractionated into 96 fractions which were then concatenated into 24 superfractions prior to LC-MS/MS analysis.
Data source location	Harvard Medical School Boston, MA USA
Data accessibility	Mass spectrometry data have been shared in the ProteomeXchange consortium via the PRIDE repository. Pride Data identification number: PXD037315 Direct URL to data: http://proteomecentral.proteomexchange.org/cgi/GetDataset?ID=PXD037315 Instructions for accessing these data: None. Data are publicly accessible.

**Value of the Data**

• This is the first dataset exploring the effect on proteome stability of Ubc4 and Ubc5.
• This dataset is of broad interest because it offers novel targets of the UPS that are involved in many biological processes. It can be useful for cell biologists, for enzymologists, and for researchers studying UPS alterations in diseases.
• The UPS is exploited as a pharmacological target (*i.e.*, for neurodegenerative diseases). These data can provide insight into the development of drugs targeting the UPS.

## 1 Data Description

**Fig. 1:**
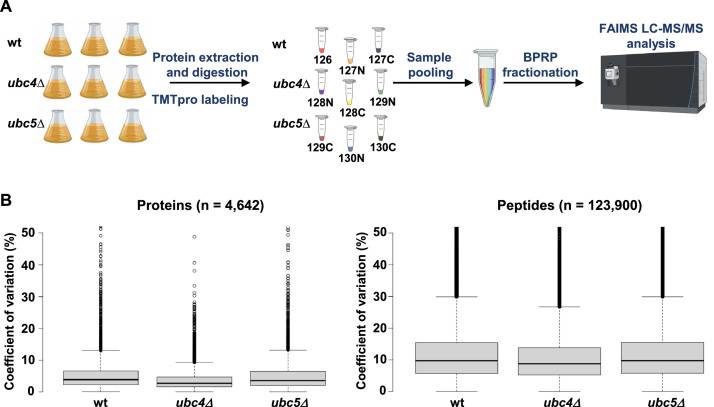
Experimental workflow and coefficient of variation among replicates. (A) Wild type (wt), *ubc4Δ* and *ubc5Δ* yeast cells were exponentially grown in triplicate, collected and lysed. Proteins were precipitated and digested with LysC and trypsin. The peptides were labeled with tandem mass tag (TMTpro) reagents and mixed 1:1. The mixed pool of peptides was fractionated by basic pH reversed-phase (BPRP) HPLC. This figure has been created, in part, using Biorender.com. (B) Left: Distribution of the coefficient of variation for the replicate measurements at the protein level. Medians of 3.8% (wt), 2.7% (*ubc4Δ*), and 3.5% (*ubc5Δ*) were calculated. Right: Distribution of the coefficient of variation for the replicate measurements at the peptide level. The medians were 9.7% (wt), 8.7% (*ubc4Δ*), and 9.7% (*ubc5Δ*).

This article contains both the RAW data files (*.raw) and the search engines results (*.mzIdentML) from a multiplexed mass spectrometry experiment in yeast cells, as well as the analyzed data. These data have been deposited in the PRIDE repository (ProteomeXchange Consortium) with the identifier PXD037315. Fig. 1 illustrates the sample preparation workflow for LC-MS/MS analysis (Fig. 1A) and also illustrates distributions of the coefficient of variation for both the proteins and peptides analyzed in the proteomics experiment (Fig. 1B). Supplemental Table 1 lists the RAW files, the fraction associated with that file, along with the compensation voltage (CV) sets used for the associated analysis. Supplemental Table 2 lists the protein identifications. Columns include: protein ID, gene symbol, description, protein group ID, the number of peptides assigned to a given protein, 9 columns of TMT signal-to-noise values and 9 columns of TMT signal-to-noise values that have been scaled to 100 across all channels.

## 2 Experimental Design, Materials and Methods

### 2.1 Yeast Strains and Growth Conditions

All yeast strains used in this study are isogenic to W303 (*ade2‐1, trp1‐1, leu2‐3112, his3‐11, 15, ura3*). Standard yeast genetics was used to generate *ubc4Δ* and *ubc5Δ* strains [1]. Cells were grown as described previously [2]. Briefly, triplicate cultures of wild type, *ubc4Δ* and *ubc5Δ* cells were grown overnight at 25 °C in YEPD medium (1% yeast extract, 2% bactopeptone, 50 mg/l adenine, 2% glucose). The next day, the cultures were diluted with fresh medium to OD_600_=0.3 (wild type and *ubc5Δ*) or OD_600_= 0.4 (*ubc4Δ)* and allowed to grow at 25 °C until mid-exponential phase (OD_600_∼1). Cells were collected by centrifugation at 2000 g for 2 min*,* rinsed with 1 ml cold water, flash-frozen in liquid nitrogen and stored at 80 °C until LC-MS/MS analysis.

### 2.2 Sample Processing for TMTpro-Proteomic Analysis

Lysis buffer (8 M urea in 200 mM EPPS, pH 8.5 plus protease and phosphatase inhibitors) was added to the yeast cell pellets. Samples were lysed by bead beating (5 cycles of 30 s). Protein concentration of each sample was determined with a BCA assay (according to manufacturer's instruction). Disulfide bonds were reduced with 5 mM TCEP (15 min), alkylated with 10 mM NEM (15 min) and the NEM was then quenched with 5 mM DTT (15 min). Alkylation and quenching were performed in the dark. 100 µg of protein from each sample was precipitated with chloroform-methanol [3], and then resuspended in 200 mM EPPS pH 8.5. Samples were digested by Lys-C (overnight at 24 °C) and trypsin (6 h at 37 °C). 1 µg of each enzyme was used per 100 µg of protein.

As preparation for TMT labeling, acetonitrile was added to a final volume of 30% to each digest. For each sample, 50 µg of peptide were labeled with 100 µg of TMTpro reagents at room temperature for one hour. The labeling scheme was as follows: wt triplicates: 126,127n,127c; *ubc4Δ* triplicates: 128n,128c,129n; *ubc5Δ* triplicates:129c,130n,130c. Upon verifying that labeling efficiency was >97% [4], the reactions were quenched by adding hydroxylamine to give a final concentration of ∼0.3% and then incubated 15 min at room temperature. The label-check was a quality control step prior to the final pooling of TMT-labeled samples. Here, we combined a small amount (1–3 µL or ∼2 µg) of each sample and analyzed it by mass spectrometry to confirm that the protein digestion was successful, if the degree of labeling was sufficient, and if the labeled samples contained approximately equal amount of peptides. During database searching, the TMTpro label was considered a variable modification at the N-terminus and at lysine residues. We then determined the labeling efficiency for the N-terminus and the lysine residues by dividing labeled N-terminal peptides by total peptides, and also the labeled lysine-terminating peptides by the total lysine-terminating peptides.

Samples were combined 1:1 so that each channel contained the same amount of starting protein. The pooled protein sample was desalted by solid phase extraction (with a 50 mg Sep-Pak column). Once dried and reconstituted in 10 mM ammonium bicarbonate/5% acetonitrile, 300 µg of peptide was fractionated by basic pH reversed-phase (BPRP) using an Agilent 1200 pump with an Agilent 300Extend C18 column (250 mm long, 2.1 mm ID, with 3.5 μm particles). Peptides were separated over a 50-min linear gradient spanning 5% to 35% acetonitrile in 10 mM ammonium bicarbonate pH 8 while using a flow rate of 0.25 mL/min. In total, 96 fractions were collected which were then concatenated to 24 superfractions which were divided into two sets of 12 non-adjacent superfractions [3]. The acidified fractions (1% formic acid, final volume) were vacuum centrifuged to near dryness. Finally, each was desalted via StageTip [4], dried again, and reconstituted in 5% acetonitrile, 5% formic acid for analysis by LC-MS/MS.

### 2.3 Mass Spectrometry Data Collection and Analysis

An Orbitrap Eclipse mass spectrometer was used to collect the data. The instrument was coupled to a Proxeon NanoLC-1200 UHPLC and to a FAIMSpro interface [5]. The sample was analyzed across a 100 μm capillary column packed with 35 cm of Accucore 150 resin (2.6 μm, 150 Å; ThermoFisher Scientific).

In total 24 RAW files were acquired. Data for the first 12 superfractions were collected using a Orbitrap Eclipse with a CV set of −40/−60/−80 V while the remaining 12 superfractions were analyzed with a CV set of −30/−50/−70 V also over a 90 min gradient. A one second TopSpeed cycle was used for each CV. The scan sequence began with an MS1 spectrum (Orbitrap analysis, resolution 60,000, 400–1600 Th, automatic gain control (AGC) target “standard”, maximum injection time “auto”). The hrMS2 stage consisted of fragmentation by higher energy collisional dissociation (HCD, normalized collision energy 36%) and analysis using the Orbitrap (AGC 200%, maximum injection time 86 ms, isolation window 0.7 Th, resolution 50,000).

The spectra, which were converted to mzXML with MSconvert [6], were searched with the Saccharomyces Genome Database (SGD; August 2021). Our forward database consisted of 6077 reviewed yeast entries and 115 common contaminants. This database was concatenated with a decoy database in which each protein sequence is reversed so as to enable the calculation of the target-decoy strategy for false discovery rate. These searches were performed using 50 ppm and 0.03 Da mass tolerances for precursors and fragments, respectively. We have traditionally used the 50 ppm mass tolerance for our Sequest and now Comet database searches. These wide mass tolerance windows were chosen to maximize sensitivity in conjunction with Comet searches and linear discriminant analysis [7]. In addition, oxidation of methionine residues (+15.995 Da) was set as a variable modification, whereas alkylation with N-ethylmaleimide at cysteine residues (+125.048 Da) and TMTpro tag modifications at peptide N-termini and lysine residues (+304.207 Da) were set as static modifications. A linear discriminant analysis was performed for PSM filtering, such that a 1% false discovery rate (FDR) for peptide-spectrum matches (PSMs) was set [8,9], after which the data were assembled further to a final protein-level FDR of 1% [10]. The manufacturer-provided isotopic impurities of each TMTpro reagent were used to correct reporter ion intensities [11]. The peptide signal-to-noise (S/N) measurements for each protein were summed. These values were normalized to equate the sum of all protein signals in each channel, so that equal protein loading was taken into account.

## Ethics Statement

This work does not include human subjects, animal experiments or data collected from social media platforms.

## CRediT authorship contribution statement

**Valentina Rossio:** Investigation, Conceptualization, Writing – original draft. **Joao A Paulo:** Resources, Conceptualization, Data curation, Software, Writing – original draft.

## Declaration of Competing Interest

The authors declare that they have no known competing financial interests or personal relationships that could have appeared to influence the work presented in this paper.

## Data Availability

Quantitative proteome dataset profiling of UBC4 and UBC5 deletion strains in Saccharomyces cerevisiae (Original data) (Pride).

## Appendix Supplementary materials

Image, application 1

Image, application 2
